# The whole chloroplast genome sequence of *Macadamia tetraphylla* (Proteaceae)

**DOI:** 10.1080/23802359.2018.1532836

**Published:** 2018-10-26

**Authors:** Jin Liu, Ying-Feng Niu, Shu-Bang Ni, Xi-Yong He, Cheng Zheng, Zi-Yan Liu, Hao-Hong Cai, Chao Shi

**Affiliations:** a Yunnan Institute of Tropical Crops, Jinghong, Xishuangbanna, PR China;; b College of Marine Science and Biological Engineering, Qingdao University of Science and Technology, Qingdao, PR China;; c Kunming Institute of Botany, Chinese Academy of Sciences, Kunming, PR China

**Keywords:** *Macadamia tetraphylla*, proteaceae, chloroplast genome

## Abstract

*Macadamia tetraphylla* (Proteaceae) is one of the two macadamia plants that are edible and of cultivated value. Only two chloroplast genomes were reported in Proteaceae so far. In this study, we report the complete chloroplast genome sequence of *M. tetraphylla*, which is the third reported chloroplast genome in Proteaceae. The chloroplast genome is 159,195 bp long and includes 113 genes. Its LSC, SSC and IR regions are 87,951, 18,748 and 26,248 bp long, respectively. Phylogenetic analysis indicates that *M. tetraphylla* was clustered with other two species of Proteaceae, the *M. integrifoia* and *M. ternifolia*.

Macadamia (*Macadamia tetraphylla*; Proteaceae) is one of the two macadamia plants that are edible and of cultivated value. Its kernel rate and oil content are low. Because of the high sugar content (Fourie and Basson 1990), processed products are prone to browning (Wall and Gentry 2005). Whereas, macadamia tree is a gracefully plant with dense branches and leaves, beautiful and aromatic flowers, solid and fine wood, and it conquers most insects and disease. Thus, it is an excellent landscaping and timber species. However, *M. tetraphylla* is becoming extinct (Shapcott and Powell 2011). Genetic analysis can determine the potential long-term viability of this species. Only two chloroplast genomes were reported in Proteaceae, the *M. integrifoia* (Nock et al. 2014) and *M. ternifolia* (Liu et al. 2017). In the study, we report the complete chloroplast genome of *M. tetraphylla*, and hope it will help restore the macadamia species to prevent the extinction of this single wild population.

The DNA material was isolated from mature leaves of a *M. tetraphylla* plant cultivated in the plant garden of Yunnan Institute of Tropical Crops (YITC), Jinghong, China and the specimen of this tree was conserved in YITC. The isolated DNA was sent to BGI Shenzhen for library construction and genome sequencing on the Illumina Hiseq 2000 Platform (Illumina, San Diego, CA) with subsequent of 3.5 Gbp reads in fastq format were obtained and used in chloroplast genome assembly. The complete chloroplast genome was annotated with Dual Organelle GenoMe Annotator (DOGMA; Wyman et al. 2004) and submitted to the Genbank (http://www.ncbi.nlm.nih.gov/) under the accession number of MH778649. A physical map of the chloroplast genome was generated by OGDRAW (http://ogdraw.mpimp-golm.mpg.de/) (Lohse et al. 2013). Our assembly of the *M. tetraphylla* resulted in a final sequence of 159,195 bp in length with no gap. The overall A-T content of the chloroplast genome was 61.2%. This chloroplast genome included a typical quadripartite structure with the Large Single Copy (LSC), Small Single Copy (SSC) and Inverted Repeat (IR) regions of 87,951, 18,748 and 26,248 long, respectively. Genome annotation showed 113 full length genes including 79 protein-coding genes, 30 tRNA genes and four rRNA genes. The genome organization, gene content and gene relative positions were almost identical to the previously reported *M. integrifoia* chloroplast genome (Nock et al. 2014). Eighteen genes were duplicated in the IR regions. Fifteen genes contained one intron, while three had two introns. Phylogenetic tree included *M. tetraphylla*, *M. integrifoia* and the other 10 representative early diverging eudicotyledons species was constructed. Maximum-likelihood (ML) analysis exhibited that *M. tetraphylla* clustered with *M. integrifoia* (Nock et al. 2014) and *M. ternifolia* (Liu et al. 2017), and other species were highly supported by a bootstrap value of 100 (Figure 1).

**Figure 1.**
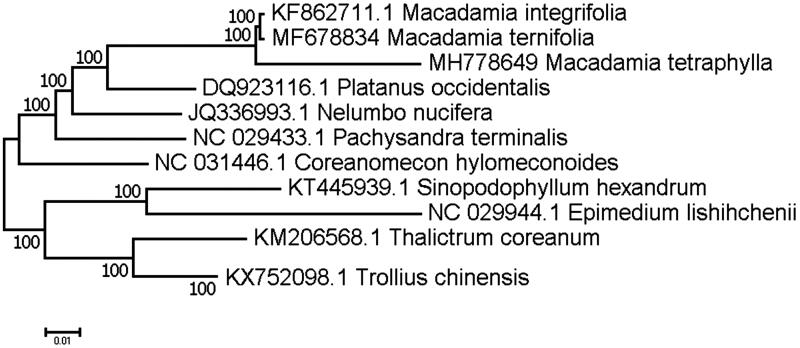
Maximum-likelihood (ML) phylogenetic tree of *M. tetraphylla* and the other 10 representative early diverging eudicotyledons species. Number above each node indicates the ML bootstrap support values.
